# Artificial intelligence in undergraduate medical education clinical skills curricula: a scoping review of implementations since 2022

**DOI:** 10.3389/fdgth.2026.1830254

**Published:** 2026-06-10

**Authors:** Birpartap S. Thind, Daryoush Javidi, Lisa M. Schwartz

**Affiliations:** 1 School of Medicine, California University of Science and Medicine, Colton, CA, United States; 2 Department of Medical Education, California University of Science and Medicine, Colton, CA, United States; 3 Department of Medical Education, California University of Science and Medicine, Colton, CA, United States

**Keywords:** artificial intelligence, clinical skills, generative AI, OSCE assessment, scoping review, undergraduate medical education, virtual patient simulation

## Abstract

**Purpose:**

To systematically identify and synthesize peer-reviewed literature describing implemented AI innovations within undergraduate medical education clinical skills curricula from January 2022 through January 2026.

**Method:**

The authors conducted a scoping review querying PubMed and Scopus, supplemented by SciSpace as an AI-assisted citation discovery tool. Eligible studies described utilizing AI to deliver the clinical skills curriculum in innovative ways (e.g., instruction in history-taking, communication, clinical reasoning, clinical documentation, OSCE/simulation assessment). We extracted data into standardized templates and thematically sorted to characterize how AI-assisted instruction was being implemented across educational objectives.

**Results:**

From 1,130 initial records, 39 studies met inclusion criteria. AI-assisted instruction clustered into eight thematic categories: LLM-Based Virtual Patient and Clinical Simulation Systems (*n* = 19), AI-Augmented OSCE and Simulation Assessment Tools (*n* = 6), Embodied and Robotic AI Clinical Simulations (*n* = 4), AI-Supported Procedural and Technical Skills Training (*n* = 3), AI-Assisted Clinical Documentation and EHR-Based Skills Training (*n* = 2), Multimodal Analytics for Skills Assessment (*n* = 2), Educator-Facing AI Case Authoring and Simulation Design Tools (*n* = 2), and AI-Supported Clinical Reasoning and Tutoring Tools (*n* = 1). Publication activity concentrated heavily in 2024–2025, with virtual patient applications representing the dominant category.

**Conclusions:**

AI implementation in clinical skills education has accelerated substantially since 2022, with large language model-powered virtual patient simulations emerging as the predominant application. Current implementations primarily position AI as a supplementary formative tool rather than a replacement for established pedagogical approaches. Robust evidence regarding long-term educational impact remains limited, indicating need for rigorous longitudinal evaluation alongside continued innovation.

## Introduction

Artificial intelligence (AI), particularly large language models (LLMs) and other generative AI (GenAI) technologies, has rapidly entered undergraduate medical education, prompting growing interest in how these tools might support core aspects of physician training. Among the most pedagogically complex domains is clinical skills education, which encompasses history-taking, physical examination, clinical reasoning, communication, and documentation. Traditionally, these competencies have relied on standardized patients, simulation exercises, and direct faculty observation, approaches that are resource-intensive and difficult to scale (1). As medical schools contend with faculty time constraints, limited standardized patient availability, and increasing demand for timely formative feedback, AI-assisted educational tools have emerged as potential complements to existing instructional models (2). Despite accelerating experimentation, the scope and nature of implemented AI innovations within undergraduate clinical skills curricula remain poorly characterized.

Interest in AI integration is driven in part by learner and educator perspectives. Medical students in the United States report strong interest in AI's relevance to their future clinical practice, yet many indicate that their institutions offer limited formal instruction or that available resources are unclear, highlighting a mismatch between learner expectations and curricular offerings (3). Medical educators likewise demonstrate generally positive attitudes toward GenAI in medical education while simultaneously expressing concerns related to reliability, bias, assessment integrity, and appropriate use (4). Together, these findings underscore the need for structured, educationally sound approaches to AI integration within medical education, rather than *ad hoc* or opportunistic adoption.

Encouragingly, national data suggests that medical schools are beginning to respond to these pressures. The Association of American Medical Colleges (AAMC) Curriculum SCOPE survey demonstrates increasing integration of AI-related content across U.S. and Canadian medical schools, with substantial growth observed over a single year (5). However, these data reflect curricular inclusion broadly and do not delineate how AI is being operationalized within specific educational domains, such as clinical skills training. As a result, it remains unclear what forms of AI are actually being implemented, for what educational purposes, and with what degree of learner engagement or evaluation.

Clinical skills curricula represent a foundational component of undergraduate medical education and are closely linked to downstream clinical performance. Prior work has demonstrated that structured, longitudinal preclinical clinical skills training can translate into measurable improvements in clerkship performance, underscoring the importance of intentional curricular design in this domain (6). Given the centrality of clinical skills to physician competence, understanding how AI is being integrated into this area of training is particularly critical.

Existing literature has begun to explore the potential applications of AI and GenAI within medical education, including narrative and conceptual reviews describing broad opportunities across educational contexts. For example, Bhuyan and colleagues outline potential GenAI applications such as simulated patient interactions, synthetic data generation, and AI-assisted instructional tools (7). However, such reviews largely remain conceptual and provide limited detail on implemented undergraduate clinical skills curricula or systematic evaluation of learner-facing educational outcomes. This highlights a persistent gap between theoretical promise and applied educational practice within clinical skills education.

To address this gap, this scoping review systematically identifies and categorizes recent AI-assisted implementations within undergraduate medical education clinical skills curricula. By characterizing how AI has been operationalized across educational functions and contexts since 2022, this review aims to provide a structured overview of current practices and inform educators, curriculum leaders, and institutions seeking to thoughtfully and responsibly integrate AI into clinical skills training.

## Method

We conducted a scoping review to characterize how AI-enabled tools have been implemented in undergraduate medical education clinical skills curricula and skills-adjacent training domains from January 1, 2022, through January 2026. This review followed the PRISMA extension for Scoping Reviews (PRISMA-ScR) guidelines. The review aimed to identify AI use as an adjunct to clinical skills training (e.g., history-taking, physical examination, communication, clinical reasoning, documentation, OSCE/simulation training and assessment) and to categorize where implementation is most concentrated across the current literature.

### Information sources and search approach

We queried two established bibliographic databases, PubMed and Scopus, and supplemented these searches with SciSpace, an AI-assisted research discovery platform. SciSpace was used as a supplementary search aid rather than a primary database source; it was selected for its capacity to perform automated forward and backward citation chaining and relevance filtering across multiple underlying databases including PubMed, Google Scholar, Semantic Scholar, and arXiv, thereby broadening the scope of literature capture beyond standard database queries. The paid tier of SciSpace was utilized to ensure comprehensive search coverage. All records retrieved via SciSpace were subject to the same title/abstract screening and eligibility criteria applied to PubMed and Scopus outputs, and citation accuracy was verified manually during full-text review.

The original query provided to SciSpace was “AI in medical education technologies implementation within clinical skills curriculum in medical schools globally,” modified to “How is AI in medical education technologies being implemented within the clinical skills curriculum across medical schools globally, since the start of 2022, to prepare medical students for providing better patient care during clinicals/clerkships and beyond?” These queries were automatically expanded into multiple, more specific research statements for targeted queries to ensure a comprehensive and manageable literature search. The system then ran both backward and forward citation chaining and relevancy filters to identify literature.

Additionally, we performed literature searches on both PubMed and Scopus using the following logic: (“artificial intelligence” OR “machine learning” OR “large language model*” OR “clinical decision support” OR “AI-assisted” OR “generative AI”) AND (“clinical skills” OR “history taking” OR “physical examination” OR “clinical reasoning” OR “documentation” OR “OSCE” OR “standardized patient*”) AND (“medical student*” OR “undergraduate medical education” OR “medical education”).

We aggregated records from each source into a master dataset. Searches were structured to capture AI modalities (e.g., artificial intelligence, machine learning, large language models, generative AI, ChatGPT) and clinical skills education contexts (e.g., clinical skills, OSCE, standardized patient, simulation, history taking, physical examination, communication skills, documentation, clinical reasoning) within undergraduate medical education/medical student populations.

### Record management and deduplication

We merged and deduplicated search outputs in two stages: (1) automated deduplication using GPT-5.2, prompted to identify exact and near-duplicate records across sources using DOI-based matching (with normalization for preprint DOI patterns) followed by title-based matching for records with missing or inconsistent DOIs, preferring peer-reviewed final versions over preprints when both were present; and (2) manual review by the primary author to identify and remove any residual duplicates.

### Screening and eligibility criteria

We performed title/abstract screening to identify candidate studies relevant to AI use in undergraduate medical education clinical skills education. We included peer-reviewed studies that described AI use as an adjunct to clinical skills teaching used for content delivery, practice, feedback, coaching, assessment, or analytics in clinical skills domains (including OSCE and simulation-based clinical skills training). We excluded records that did not pass title/abstract screening for relevance to undergraduate medical education clinical skills implementation, non-English records, inaccessible full texts/conference abstracts, duplicates, and preprints (per above).

### Data extraction and thematic synthesis

For each included study, we extracted structured descriptors and qualitative summaries into a standardized spreadsheet template (year, title, authors, DOI, abstract). Thematic synthesis was led by the primary author and reviewed iteratively with co-authors throughout the categorization process. Themes were derived inductively from the extracted data rather than from a predetermined framework, with initial codes developed by grouping studies according to their primary educational function, AI modality, and implementation context. Similar codes were progressively consolidated into broader thematic categories through iterative discussion among the author team, with disagreements resolved by consensus. Final categories were designed to be reader-facing and mutually interpretable across heterogeneous implementations. Each of the 39 included studies was assigned one primary overarching theme to enable frequency-based reporting of where the bulk of implementation activity is occurring; studies that could reasonably span multiple categories were assigned to the theme most reflective of their primary educational objective.

## Results

**Figure 1:**
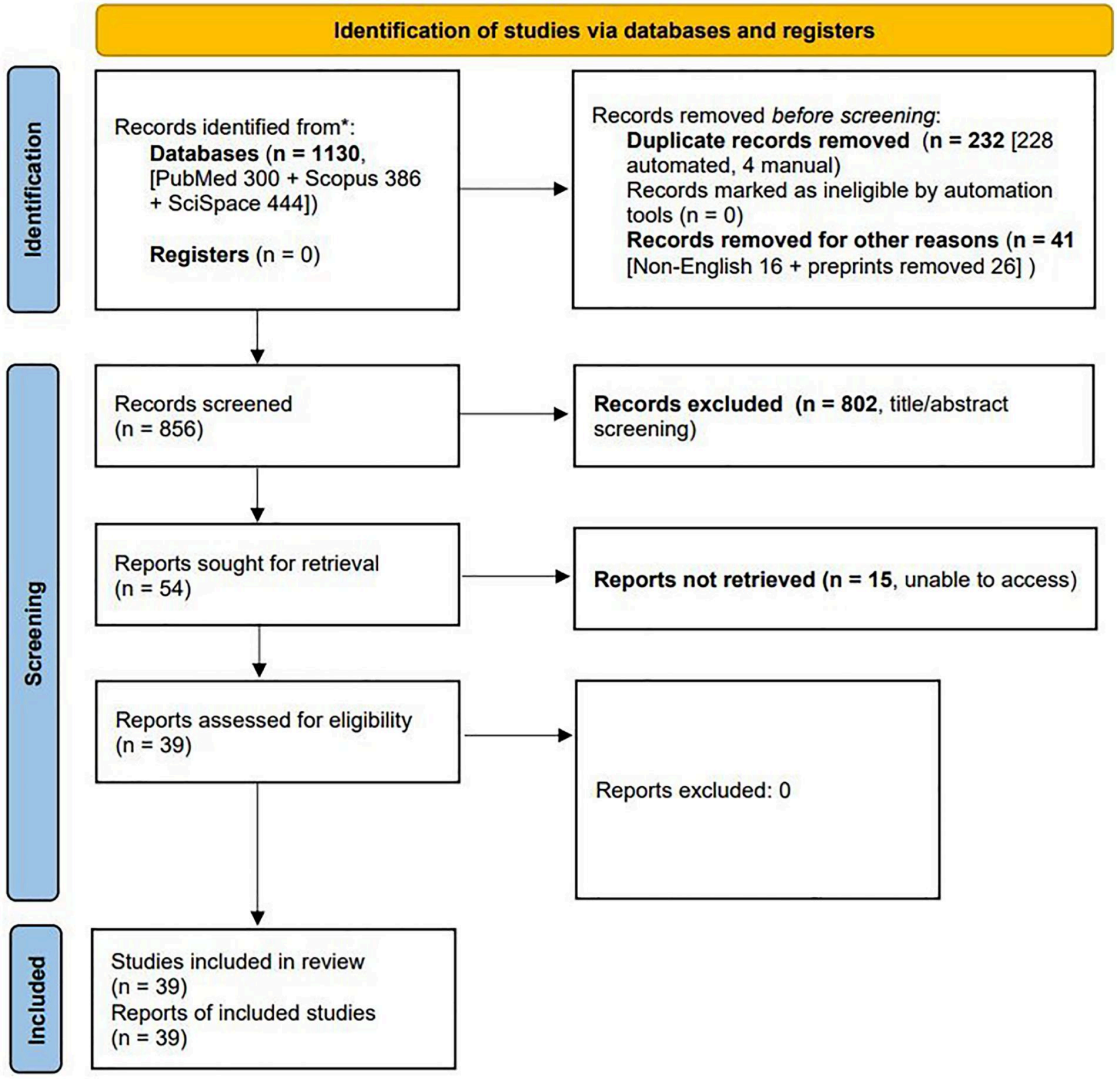
PRISMA flow diagram of study selection. Flowchart depicting the systematic screening process from initial database searches (*n* = 1,130) through deduplication, eligibility screening, and final inclusion (*n* = 39). Records were excluded for non-English language, duplicate status, preprint format, failure to meet relevance criteria during title/abstract screening, and inaccessible full text. PRISMA 2020 flow diagram for new systematic reviews which included searches of databases and registers only. *SciSpace results incorporated the platform's automated forward/backward citation chaining and relevance filtering. SciSpace reported identifying 24 additional records via citation chaining; the final SciSpace export used in this review contained 444 records. Adapted from “PRISMA 2020 flow diagram template for systematic reviews” by Page et al., licensed under CC BY 4.0.

Across PubMed, Scopus, and SciSpace, we identified 1,130 records (PubMed: 300; Scopus: 386; SciSpace: 444). After automated deduplication (228 removed), 902 unique records remained. We then removed non-English records (*n* = 16), manually removed residual duplicates (*n* = 4), and removed preprints (*n* = 26). During title/abstract screening, 802 records were excluded for not meeting relevance criteria. Full text was unavailable for 15 records. This process yielded 39 included studies describing AI implementation in undergraduate medical education clinical skills curricula or closely skills-adjacent domains (Figure 1; Table 1).

**Table 1:** AI implementation studies within clinical skills curricula (*N* = 39).

Year	Title	Authors	DOI	Theme
2026	Applying multimodal learning analytics to naturalistic recordings of clinical simulations: Towards an accurate and scalable pipeline for automated feedback generation	Popov, V.; Nguyen, S.; Ochoa, X.	10.1016/j.learninstruc.2025.102267	Multimodal Analytics for Skills Assessment
2025	Applying state-of-the-art artificial intelligence to grading in simulation-based education: assessment, feedback, and ROI	Campbell, K.K.; Holcomb, M.J.; Vedovato, S.; et al.	10.1007/s44163-025-00417-3	AI-Augmented OSCE/Simulation Assessment & Grading
2025	Evaluating the Effectiveness of ChatGPT Versus Human Proctors in Grading Medical Students’ Post-OSCE Notes	Thomas, K.; Szalacha, L.; Hanna, K.; Anibal, J.; Petrilli, J.	10.22454/fammed.2025.954255	AI-Augmented OSCE/Simulation Assessment & Grading
2025	Application of Artificial Intelligence as an Aid for the Correction of the Objective Structured Clinical Examination (OSCE)	Luordo, D.; Torres-Arrese, M.; Tristán Calvo, C.; et al.	10.3390/app15031153	AI-Augmented OSCE/Simulation Assessment & Grading
2025	Is AI the future of evaluation in medical education?? AI vs. human evaluation in objective structured clinical examination	Tekin M, Yurdal MO, Toraman Ç, Korkmaz G, Uysal İ.	10.1186/s12909-025-07241-4	AI-Augmented OSCE/Simulation Assessment & Grading
2025	Effectiveness of traditional, artificial intelligence-assisted, and virtual reality training modalities for focused cardiac ultrasound skill acquisition: a randomised controlled study	Lau YH, Acharyya S, Wee CWL, Xu H, Saclolo RP, Cao K, Fong WK.	10.1186/s13089-025-00469-7	AI-Supported Procedural and Technical Skills Training
2025	Real-world implementation of an AI learning tool-MetaGP-Edu in medical education: A multi-center cohort study	Sun, Y.; Liu, F.	10.1016/j.compedu.2025.105388	AI-Supported Clinical Reasoning and Tutoring Tools
2025	Virtual Patient Simulations Using Social Robotics Combined With Large Language Models for Clinical Reasoning Training in Medical Education: Mixed Methods Study	Borg A, Georg C, Jobs B, Huss V, Waldenlind K, Ruiz M, Edelbring S, Skantze G, Parodis I.	10.2196/63312	Embodied and Robotic AI Clinical Simulations
2025	AI-Enhanced Social Robotic Versus Computer-Based Virtual Patients for Clinical Reasoning Training in Medical Education: Observational Crossover Cohort Study	Borg A, Schiött J, Ivegren W, et al.	10.2196/82541	Embodied and Robotic AI Clinical Simulations
2025	Physical Examination Identification in Medical Education Videos: Zero-Shot Multimodal AI With Temporal Sequence Optimization Study	Kang S, Holcomb M, Hein D, Shakur AH, Dalton T, Jamieson A.	10.2196/76586	Multimodal Analytics for Skills Assessment
2025	Creating virtual patients using large language models: scalable, global, and low cost	Cook, D.A.	10.1080/0142159x.2024.2376879	Educator-Facing AI Case Authoring and Simulation Design Tools
2025	Applying ChatGPT to plan and create a realistic collection of virtual patients for clinical reasoning training	Fąferek J, Kononowicz AA, Bogutska N, et al.	10.1186/s12909-025-08006-9	Educator-Facing AI Case Authoring and Simulation Design Tools
2025	Development of a GPT-4-Powered Virtual Simulated Patient and Communication Training Platform for Medical Students to Practice Discussing Abnormal Mammogram Results With Patients: Multiphase Study	Weisman D, Sugarman A, Huang YM, Gelberg L, Ganz PA, Comulada WS.	10.2196/65670	LLM-Based Virtual Patient and Clinical Simulation Systems
2025	Understanding the Role of Large Language Model Virtual Patients in Developing Communication and Clinical Skills in Undergraduate Medical Education	Sheth, U.; Lo, M.; McCarthy, J.; et al.	10.3390/ime4040039	LLM-Based Virtual Patient and Clinical Simulation Systems
2025	Beyond Traditional Simulation: An Exploratory Study on the Effectiveness and Acceptability of ChatGPT-4o Advanced Voice Mode for Communication Skills Practice Among Medical Students	Mukadam A, Suresh S, Jacobs C.	10.7759/cureus.84381	LLM-Based Virtual Patient and Clinical Simulation Systems
2025	Comparing AI chatbot simulation and peer role-play for OSCE preparation: a pilot randomized controlled trial	Lee HY, Kim J, Choi H, Bae H, Jeong A, Choi S, Kim JH, Kim CE.	10.1186/s12909-025-08308-y	LLM-Based Virtual Patient and Clinical Simulation Systems
2025	ECOSBot: a multicenter validation pilot study of a generative AI tool for OSCE-based nephrology training	Bentegeac R, Florens N, Maanaoui M, et al.	10.1093/ckj/sfaf308	LLM-Based Virtual Patient and Clinical Simulation Systems
2025	Simulated patient systems powered by large language model-based AI agents offer potential for transforming medical education	Yu H, Zhou J, Li L, et al.	10.1038/s43856-025-01283-x	LLM-Based Virtual Patient and Clinical Simulation Systems
2025	Clinical Simulation with ChatGpt: A Revolution in Medical Education?	Peralta Ramirez AA, Trujillo López S, Navarro Armendariz GA, et al.	10.1080/28338073.2025.2525615	LLM-Based Virtual Patient and Clinical Simulation Systems
2025	Feasibility study of using GPT for history-taking training in medical education: a randomized clinical trial	Wang Z, Fan TT, Li ML, Zhu NJ, Wang XC.	10.1186/s12909-025-07614-9	LLM-Based Virtual Patient and Clinical Simulation Systems
2025	DIALOGUE: A Generative AI-Based Pre-Post Simulation Study to Enhance Diagnostic Communication in Medical Students Through Virtual Type 2 Diabetes Scenarios	Suárez-García RX, Chavez-Castañeda Q, Orrico-Pérez R, et al.	10.3390/ejihpe15080152	LLM-Based Virtual Patient and Clinical Simulation Systems
2025	Using Large Language Models to Simulate History Taking: Implications for Symptom-Based Medical Education	Huh, C.Y., Lee, J., Kim, G. et al.	10.3390/info16080653	LLM-Based Virtual Patient and Clinical Simulation Systems
2025	Integrating large language model-based agents into a virtual patient chatbot for clinical anamnesis training	Laverde N, Grévisse C, Jaramillo S, Manrique R.	10.1016/j.csbj.2025.05.025	LLM-Based Virtual Patient and Clinical Simulation Systems
2025	Assessing ChatGPT's Capability as a New Age Standardized Patient: Qualitative Study	Cross J, Kayalackakom T, Robinson RE, et al.	10.2196/63353	LLM-Based Virtual Patient and Clinical Simulation Systems
2025	Development and Validation of a Large Language Model-Based System for Medical History-Taking Training: Prospective Multicase Study on Evaluation Stability, Human-AI Consistency, and Transparency	Liu Y, Shi C, Wu L, Lin X, Chen X, Zhu Y, Tan H, Zhang W.	10.2196/73419	LLM-Based Virtual Patient and Clinical Simulation Systems
2025	LLM-Based Clinical History Taking System: A Persona-Driven Approach	Choi D, Jung Y, Kim J, Oh N, Oh H, Lee S, Seo J, Kim T.	10.3233/shti251254	LLM-Based Virtual Patient and Clinical Simulation Systems
2024	Validity evidence supporting clinical skills assessment by artificial intelligence compared with trained clinician raters	Johnsson V, Søndergaard MB, Kulasegaram K, et al.	10.1111/medu.15190	AI-Augmented OSCE/Simulation Assessment & Grading
2024	Assessing the Ability of a Large Language Model to Score Free-Text Medical Student Clinical Notes: Quantitative Study	Burke HB, Hoang A, Lopreiato JO, et al.	10.2196/56342	AI-Augmented OSCE/Simulation Assessment & Grading
2024	ChatGPT's Ability to Assist with Clinical Documentation: A Randomized Controlled Trial	Baker HP, Dwyer E, Kalidoss S, Hynes K, Wolf J, Strelzow JA.	10.5435/jaaos-d-23-00474	AI-Assisted Clinical Documentation and EHR-Based Skills Training
2024	Creating Virtual Patients using Robots and Large Language Models: A Preliminary Study with Medical Students	Borg, A.; Parodis, I.; Skantze, G.	10.1145/3610978.3640592	Embodied and Robotic AI Clinical Simulations
2024	Enhancing clinical reasoning skills for medical students: a qualitative comparison of LLM-powered social robotic versus computer-based virtual patients within rheumatology	Borg A, Jobs B, Huss V, et al.	10.1007/s00296-024-05731-0	Embodied and Robotic AI Clinical Simulations
2024	OSCEai: personalized interactive learning for undergraduate medical education	Eddie Guo, Rashi Ramchandani, Ye-Jean Park, Mehul Gupta	10.36834/cmej.79220	LLM-Based Virtual Patient and Clinical Simulation Systems
2024	Large language models improve clinical decision making of medical students through patient simulation and structured feedback: a randomized controlled trial	Brügge E, Ricchizzi S, Arenbeck M, et al.	10.1186/s12909-024-06399-7	LLM-Based Virtual Patient and Clinical Simulation Systems
2024	A Generative Pretrained Transformer (GPT)-Powered Chatbot as a Simulated Patient to Practice History Taking: Prospective, Mixed Methods Study	Holderried F, Stegemann-Philipps C, Herschbach L, et al.	10.2196/53961	LLM-Based Virtual Patient and Clinical Simulation Systems
2024	Enhancing Medical Interview Skills Through AI-Simulated Patient Interactions: Nonrandomized Controlled Trial	Yamamoto A, Koda M, Ogawa H, Miyoshi T, Maeda Y, Otsuka F, Ino H.	10.2196/58753	LLM-Based Virtual Patient and Clinical Simulation Systems
2024	A Language Model-Powered Simulated Patient With Automated Feedback for History Taking: Prospective Study	Holderried F, Stegemann-Philipps C, Herrmann-Werner A, Festl-Wietek T, Holderried M, Eickhoff C, Mahling M.	10.2196/59213	LLM-Based Virtual Patient and Clinical Simulation Systems
2023	AI in Surgical Curriculum Design and Unintended Outcomes for Technical Competencies in Simulation Training	Ali M. Fazlollahi, Recai Yilmaz, Alexander Winkler-Schwartz, et al.	10.1001/jamanetworkopen.2023.34658	AI-Supported Procedural and Technical Skills Training
2022	Effect of Artificial Intelligence Tutoring vs Expert Instruction on Learning Simulated Surgical Skills Among Medical Students: A Randomized Clinical Trial	Fazlollahi AM, Bakhaidar M, Alsayegh A, et al.	10.1001/jamanetworkopen.2021.49008	AI-Supported Procedural and Technical Skills Training
2022	Intelligent virtual case learning system based on real medical records and natural language processing	Wang M, Sun Z, Jia M, Wang Y, Wang H, Zhu X, Chen L, Ji H.	10.1186/s12911-022-01797-7	AI-Assisted Clinical Documentation and EHR-Based Skills Training

Table 1 contains complete data extraction for all 39 included studies, organized by year, title, authors, DOI, and assigned thematic category.

### Publication year distribution

Included studies were predominantly recent, with the majority published in 2025 (*n* = 25), followed by 2024 (*n* = 10). Earlier years contributed fewer implementation studies (2022: *n* = 2; 2023: *n* = 1; 2026: *n* = 1), consistent with rapid acceleration of AI use as an adjunct to clinical skills teaching published in the last 12–18 months.

### Overarching themes

AI-assisted instruction clustered into eight overarching themes, with a clear dominance of LLM-Based Virtual Patient and Clinical Simulation Systems (Table 2).

**Table 2:** Overarching thematic categories of AI implementation.

Theme	*n*	Definition
LLM-Based Virtual Patient and Clinical Simulation Systems	19	AI-driven simulated patient interactions and simulation-style practice to develop core clinical skills, most commonly history-taking and communication skills, and in some cases clinical reasoning or diagnostic communication.
AI-Augmented OSCE/Simulation Assessment & Grading	6	AI applications to score OSCE performances or simulation outputs (e.g., interview scoring, note grading, evaluator augmentation), reflecting interest in automating or scaling assessment and feedback.
Embodied and Robotic AI Clinical Simulations	4	Implementations integrating AI with embodied interfaces such as social robotics or immersive experiences, using conversational AI/LLMs to deliver interactive clinical encounters beyond text-only chat.
AI-Supported Procedural and Technical Skills Training	3	AI feedback or AI-enhanced simulation for procedural/technical skills training (e.g., surgical simulation, ultrasound/technical task training).
AI-Assisted Clinical Documentation and EHR-Based Skills Training	2	AI tools that support learners in translating simulated or standardized patient encounters into clinical documentation, emphasizing documentation as a clinical skill.
Multimodal Analytics for Skills Assessment	2	Multimodal learning analytics (e.g., video/audio-based detection or analysis) to assess clinical skills performance signals beyond text.
Educator-Facing AI Case Authoring and Simulation Design Tools	2	Faculty-facing AI tools used to design, generate, or curate virtual patient cases or simulation scenarios, enabling scalable content creation.
AI-Supported Clinical Reasoning and Tutoring Tools	1	AI systems embedded within coursework to scaffold clinical reasoning, decision-making, or learning longitudinally, rather than simulating patient encounters.

AI, artificial intelligence; GenAI, generative artificial intelligence; LLM, large language model; OSCE, objective structured clinical examination.

### Temporal pattern by theme

**Figure 2:**
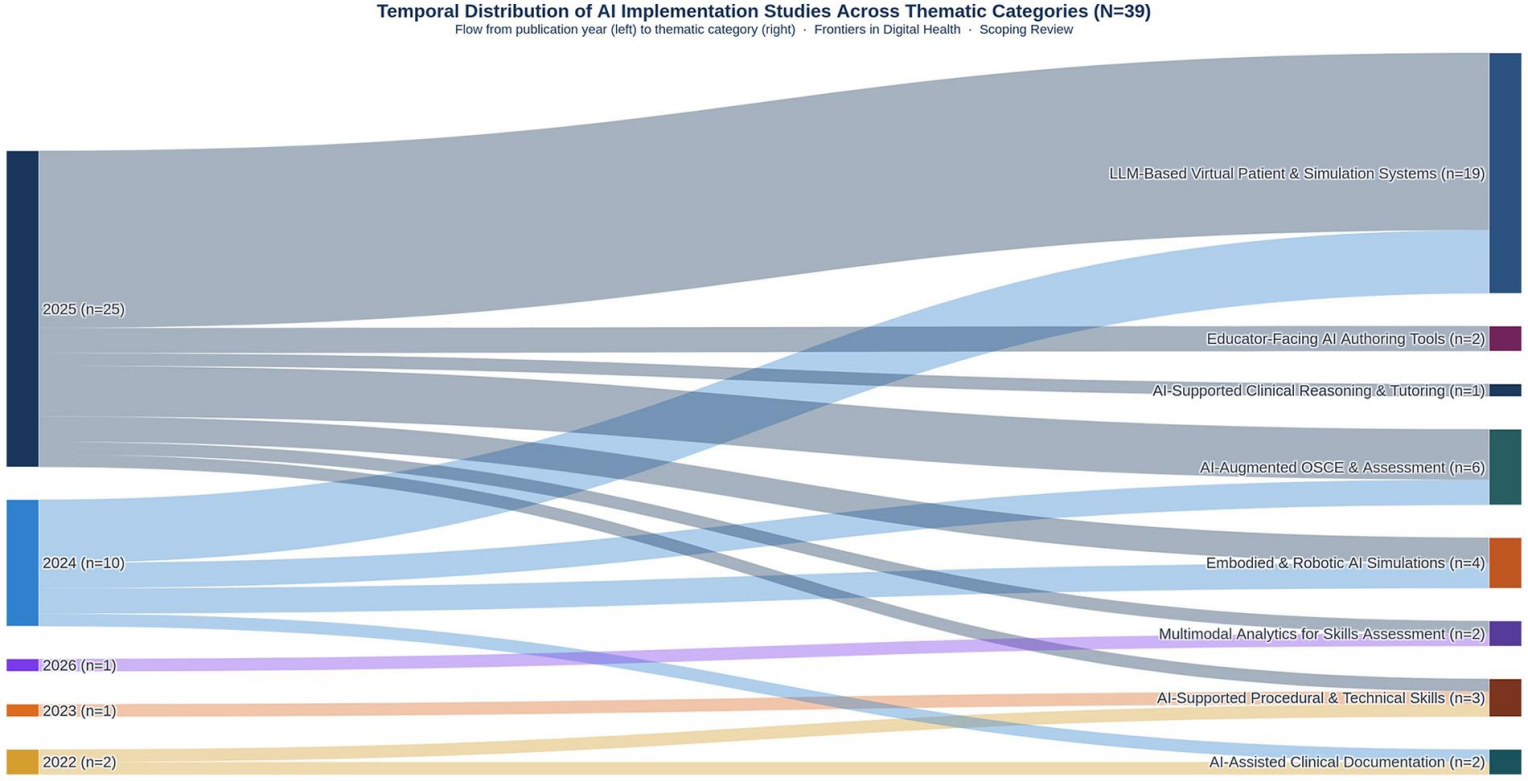
Sankey diagram illustrating the temporal distribution of included studies across thematic categories (*N* = 39). Each node on the left represents a publication year (2022 through January 2026); each node on the right represents one of eight AI implementation themes. Band width is proportional to the number of studies flowing from a given year into each thematic category. The diagram demonstrates the concentration of publication activity in 2024–2025, with a single study identified from January 2026, and the dominance of LLM-Based Virtual Patient and Clinical Simulation Systems across all years.

The “LLM-Based Virtual Patient and Clinical Simulation Systems” theme was heavily concentrated in 2025, reflecting rapid growth in LLM-enabled conversational simulations. “AI-Augmented OSCE and Simulation Assessment Tools” also increased from 2024 to 2025. The “Educator-Facing AI Case Authoring and Simulation Design Tools” appeared exclusively in 2025 within the included set, suggesting a newer wave of educator-facing tooling following earlier learner-facing simulations. These temporal patterns are summarized visually in Figure 2.

## Discussion

This scoping review synthesizes 39 peer-reviewed studies describing implemented or piloted AI-enabled instruction within undergraduate medical education clinical skills curricula since 2022. Collectively, these studies demonstrate a clear shift from conceptual discussions of AI in medical education toward early stage yet tangible educational applications. Across the included literature, AI is used as an adjunct to clinical skills teaching clustered into eight overarching functional themes. While “LLM-Based Virtual Patient and Clinical Simulation Systems” constituted the largest group, the studies collectively reflect a heterogeneous implementation landscape spanning learner-facing practice tools, assessment augmentation systems, educator-facing authoring platforms, and exploratory multimodal or embodied simulations (40, 50, 51). This diversity suggests that clinical skills education has emerged as a particularly fertile domain for applied AI, likely because its core activities, such as history-taking, communication, examination, documentation, and OSCE-style performance, are structured, observable, and interaction-based.

For purposes of this review, implementation outcomes are defined as feasibility, acceptability, and operational deployment of AI-enabled tools within clinical skills educational settings, whereas educational outcomes refer to measurable changes in learner performance, skill acquisition, retention, or transfer to clinical practice. The majority of included studies address implementation outcomes; evidence pertaining to educational outcomes, particularly longitudinal skill retention and clinical transfer, remains limited across the literature.

Representative implementations include text-based LLM chatbots such as OSCEai and ECOSBot for history-taking and OSCE preparation, voice-enabled systems such as ChatGPT-4o Advanced Voice Mode for communication practice, social robotic platforms for embodied clinical simulation, multimodal analytics pipelines for automated performance feedback, and educator-facing tools such as those described by Cook and Fąferek et al. for scalable virtual patient case authoring (8–17).

### Virtual patients as the predominant mode of AI integration

More than half of the included studies focused on AI-powered virtual patients or generative clinical simulations, making this the most mature and widely implemented category to date, as reflected by publication volume, diversity of institutional contexts, and frequency of curricular deployment across the included literature (14, 17–19, 41–43, 46–49, 52). These systems are primarily designed to support student practice in history-taking, communication, and clinical reasoning in low-stakes or formative contexts, often outside scheduled curricular time.

The virtual patient literature does not reflect a single uniform approach. Several implementations rely on text-based LLM chat interfaces to simulate medical interviews and clinical anamnesis (20–22). Others integrate voice-based interaction, allowing learners to practice spoken communication and conversational flow (23). A subset of studies employ persona-driven, scripted, or constraint-based architectures, explicitly shaping LLM outputs to maintain role fidelity, suppress excessive verbosity, or reduce hallucinated content (24).

Several virtual patient systems are clinically domain-specific, targeting focused communication scenarios such as abnormal mammogram disclosure, diabetes counseling, nephrology interviews, or oncology discussions (25–27). The reviewed studies collectively indicate that effective AI integration appears to depend less on general-purpose conversational capability and more on alignment between simulation design and defined learning objectives.

Across studies, feasibility and learner acceptability were consistently reported, and some demonstrated short-term improvements in performance or structured task completion. However, few studies assessed longitudinal skill retention or transfer to real patient encounters, reinforcing that current virtual patient systems function primarily as supplementary rehearsal tools rather than replacements for standardized patient programs.

### AI-augmented assessment and feedback in OSCEs and simulation

A second prominent cluster involved AI-supported assessment and grading in OSCEs and simulation-based education (28–31, 44, 45). These studies explored applications including automated interview scoring, post-OSCE clinical note grading, checklist alignment, and feedback generation.

All assessment-focused implementations positioned AI as an augmentative system, intended to support faculty evaluators rather than replace them. Several studies explicitly examined human-AI concordance, reliability, or consistency, reflecting awareness of validity concerns inherent to assessment innovation. Most applications were deployed in formative or exploratory contexts, with limited use in high-stakes summative evaluation.

This cautious implementation approach aligns with findings from educator perception studies, which report optimism about AI's potential alongside concerns regarding reliability, bias, transparency, and assessment integrity (4). Taken together, the reviewed literature indicates that assessment may represent a high-impact but carefully governed entry point for AI integration, though further validation in operational educational settings is needed before broader deployment.

### Embodied and robotic AI clinical simulations

A smaller but conceptually significant subset of studies explored embodied and multimodal AI systems, including social robotics and multimodal learning analytics (8–11). Embodied implementations integrated LLM-driven conversational agents with robotic or physical interfaces to approximate nonverbal communication, turn-taking, and presence. Multimodal analytics approaches analyzed audio, video, or temporal performance data to generate automated feedback beyond text-based inputs.

The embodied and robotic AI simulation studies included in this review largely originate from a single research group employing a shared social robotics platform across multiple iterations. While this limits generalizability and indicates that embodied AI remains at an early adoption stage, the presence of successive studies using related methodologies suggests a coherent and maturing research program rather than isolated proof-of-concept demonstrations. Collectively, these studies highlight the potential of embodied AI systems to augment communicative realism and interactional fidelity beyond text-based simulations, while also underscoring the resource requirements that may constrain widespread implementation.

### Educator-facing AI and the shift toward content authoring

An underrecognized but potentially transformative finding of this review is the emergence of educator-facing AI tools, particularly for virtual patient and case authoring (12, 13). Unlike learner-facing chatbots, these systems support faculty in generating, curating, and scaling collections of clinical scenarios aligned with curricular goals.

Such tools may have an outsized impact on institutions with limited access to standardized patient resources or instructional design infrastructure. Beyond content creation, these tools have potential implications for faculty workload reduction, curriculum standardization across sites, and localization of clinical scenarios to institution-specific patient populations and learning objectives. By lowering barriers to scenario creation and localization, educator-facing AI systems could democratize access to simulation-based learning. Although currently represented by few studies, the reviewed evidence suggests this category may play a central role in the sustainability and equity of AI integration as adoption expands.

### Emerging directions informed by background AI literature

Beyond the AI implementations captured in the 39 included studies, background and related literature highlights several emerging directions that intersect with clinical skills education but are not yet fully realized within undergraduate medical education curricula. Conceptual, policy-oriented, and early implementation work emphasizes the potential for AI to support adaptive learning pathways, longitudinal competency tracking, and programmatic assessment, particularly when integrated across curricular phases rather than deployed as isolated instructional tools (8, 9). These directions align with competency-based medical education frameworks but remain only partially operationalized in current undergraduate clinical skills implementations, particularly in assessment and programmatic evaluation.

Recent benchmarking work further contextualizes these emerging directions by examining the technical feasibility of AI-assisted assessment in controlled settings. In evaluations of large language models for interview-based OSCE scoring using expert-derived rating scales, models demonstrated high intra-rater reliability and moderate accuracy relative to expert consensus, with performance varying by prompt strategy and assessment item (32). Importantly, these studies did not involve learner-facing deployment, curricular integration, or evaluation of educational outcomes, highlighting the distinction between technical readiness and operational implementation within undergraduate clinical skills education.

Adjacent literature identifies applications extending beyond the dominant virtual patient paradigm. These include embodied or immersive simulation environments (including extended or virtual reality–based platforms incorporating AI), AI-enhanced video review and structured reflective practice, diagnostic interpretation and procedural skills training (e.g., ECG/POCUS-focused modules), generative visual content creation for instruction, customizable institution-specific virtual patient frameworks, and AI-assisted transcription or documentation workflows for simulated encounters (8–10, 33–35). Foundational elements of several of these approaches are represented among the included studies, for example, AI-supported documentation as a clinical skill and multimodal performance analysis. Yet most current implementations remain task-specific or short-term rather than fully integrated or longitudinal educational systems.

More expansive realizations of these approaches were typically excluded from the present review because they lacked direct undergraduate curricular implementation, focused primarily on postgraduate or procedural training, remained at a prototype or proof-of-concept stage, or did not report formal learner-facing educational outcomes. As such, the existing evidence base reflects an early and selective subset of a broader design space for AI-enabled clinical skills education, rather than a comprehensive representation of all proposed applications.

Background literature consistently highlights unresolved governance and ethical considerations associated with these emerging directions, including data privacy, bias mitigation, transparency, and appropriate disclosure to learners (9, 10, 35). The limited empirical examination of these issues within current implementation studies suggests that future work must address not only technical feasibility and educational effectiveness, but also the institutional and ethical frameworks necessary to support responsible integration of AI into clinical skills curricula.

### Bridging the implementation-evidence gap

Despite the rapid growth in AI implementation activity documented in this review, robust evidence regarding long-term educational impact and transfer to authentic clinical performance remains limited. Most included studies employed feasibility, pilot, or short-term pre-post designs, with relatively few randomized controlled trials and minimal longitudinal follow-up (Table 1) (36). This pattern mirrors early adoption phases observed in prior educational innovations, including simulation-based training and standardized patient programs, in which implementation preceded rigorous outcome evaluation. Within the included studies, AI is most commonly positioned as a formative or supplementary tool rather than a replacement for established instructional or assessment modalities, and the existing evidence base does not yet permit definitive conclusions regarding equivalence or superiority relative to traditional approaches.

At the same time, recent studies suggest a gradual shift from feasibility-focused experimentation toward questions of design, integration, evaluation, and governance within existing curricula (37). Until more methodologically rigorous and longitudinal studies are conducted, evidence-informed guidance for widespread curricular integration will remain constrained.

### Equity, ethics, and curriculum governance

As AI-enabled clinical skills tools move from pilot implementations toward broader curricular adoption, equity, ethics, and governance considerations become increasingly salient. On one hand, scalable virtual simulations and AI-supported feedback mechanisms may expand access to clinical skills practice for learners in geographically remote or resource-constrained settings. On the other hand, uneven institutional capacity to adopt, customize, and sustain AI systems risks exacerbating disparities across medical schools and training environments.

Ethical considerations such as transparency of AI use, appropriate disclosure to learners, data privacy, and the potential propagation of bias are frequently raised in conceptual and policy-oriented literature but are rarely evaluated empirically within implementation studies (4, 7, 38). The limited treatment of these issues within the 39 included studies suggests that governance frameworks have lagged behind technical development. As AI becomes more embedded within undergraduate clinical skills curricula, the reviewed literature collectively points to the need for intentional institutional oversight, faculty engagement, and ethical guardrails to support responsible and equitable adoption.

Concrete governance measures warranting consideration include structured disclosure statements informing learners when AI is involved in instruction or assessment, audit mechanisms for AI-generated feedback, data retention and anonymization policies governing student interaction logs, and systematic bias monitoring frameworks to identify differential performance across learner subgroups.

### Limitations

This scoping review has several limitations. Despite a comprehensive search strategy, relevant AI-assisted implementations may have been missed, particularly non-English publications, institution-specific initiatives, or emerging conference outputs. In addition, the literature search was completed in January 2026, and the rapid pace of innovation in this field means that new studies may have appeared since that date.

Consistent with established scoping review methodology, formal risk-of-bias assessment was not performed, as the primary objective was to map the range and characteristics of existing implementations rather than evaluate intervention effectiveness. Additionally, this review was not prospectively registered in an open repository, which is acknowledged as a limitation with respect to methodological transparency and reproducibility (39). Thematic categorization required interpretive judgment, and some implementations could reasonably span multiple thematic categories. Publication bias may favor studies reporting successful or positive implementations over null or negative findings.

## Conclusions

This review documents a rapidly expanding body of implemented AI applications within undergraduate clinical skills education. LLM-powered virtual patient systems dominate current practice, reflecting pragmatic responses to scalability constraints, while AI-assisted assessment, embodied simulations, multimodal analytics, and educator-facing authoring tools signal a diversifying ecosystem.

Although evidence for long-term impact remains nascent, AI is no longer peripheral to clinical skills education. Rather than replacing established pedagogical methods, current implementations position AI as a complementary instructional resource requiring rigorous evaluation, thoughtful governance, and continued scholarly inquiry. This review provides a structured foundation to inform future research, curriculum design, and institutional decision-making in this rapidly evolving domain.
